# Star-Shaped Fe_3-x_O_4_-Au Core-Shell Nanoparticles: From Synthesis to SERS Application

**DOI:** 10.3390/nano10020294

**Published:** 2020-02-10

**Authors:** Thi Thuy Nguyen, Stephanie Lau-Truong, Fayna Mammeri, Souad Ammar

**Affiliations:** 1 Université de Paris, ITODYS, CNRS, UMR 7086, 15 rue J-A de Baïf, 75013 Paris, France; stephanie.lau@univ-paris-diderot.fr (S.L.-T.); ammarmer@univ-paris-diderot.fr (S.A.); 2 Department of Advanced Materials Science and Nanotechnology, University of Science and Technology of Hanoi, Vietnam Academy of Science and Technology, 18 Hoang Quoc Viet, Hanoi 10000, Vietnam; 3 Graduate University of Science and Technology, Vietnam Academy of Science and Technology, 18 Hoang Quoc Viet, Cau Giay, Hanoi 10000, Vietnam; 4 Institute of Physics, Vietnam Academy of Science and Technology, 18 Hoang Quoc Viet, Cau Giay, Hanoi 10000, Vietnam

**Keywords:** SERS, iron oxide, gold, star-shaped, seed-mediated growth

## Abstract

In this work, the preparation of magneto-plasmonic granular nanostructures and their evaluation as efficient substrates for magnetically assisted surface enhanced Raman spectroscopy (SERS) sensing are discussed. These nanostructures consist of star-shaped gold Au shell grown on iron oxide Fe_3-x_O_4_ multicores. They were prepared by seed-mediated growth of anisotropic, in shape gold nanosatellites attached to the surface of polyol-made iron oxide polycrystals. In practice, the 180 nm-sized spherical iron oxide particles were functionalized by (3-aminopropyl) triethoxysilane (APTES) to become positively charged and to interact, in solution, with negatively charged 2 nm-sized Au single crystals, leading to nanohybrids. These hybrids acted subsequently as nucleation platforms for the growth of a branched gold shell, when they were contacted to a fresh HAuCl_4_ gold salt aqueous solution, in the presence of hydroquinone, a reducing agent, for an optimized nominal weight ratio between both the starting hybrids and the gold salt. As expected, the resulting nanocomposites exhibit a high saturation magnetization at room temperature and a rough enough plasmonic surface, making them easily attracted by a lab. magnet, while exhibiting a great number of SERS hot spots. Preliminary SERS detection assays were successfully performed on diluted aqueous thiram solution (10^−8^ M), using these engineered substrates, highlighting their capability to be used as chemical trace sensors.

## 1. Introduction

Star-shaped Fe_3-x_O_4_-Au core-satellites and core-shell nanoparticles provide interesting magnetic and optical properties, making them appealing to broaden advances in magnetically assisted surface-enhanced Raman spectroscopy (SERS) applications [1,2,3,4,5,6,7]. On one hand, the anisotropic, in shape continuous or discontinuous gold structure with a thickness of less than 100 nm produces local high electric field enhancement, proceeding at the end of the gold tips and/or at their junctions (hot spots), for which the concentration of free electrons is high. So, when an external electrical field, like a laser light, is applied, the surface plasmonic resonance becomes more effective, and then SERS sensing activity more useful [8]. On the other hand, the magnetic properties of the iron oxide component offer the opportunity to selectively enrich the target molecules and to separate the SERS substrate from the whole matrix magnetically [9,10,11,12,13], for analysis. In this way, the analytical detection procedure becomes simple and the cyclical use of the substrate is facilitated. 

Focusing on the growth of a branched gold shell on an iron oxide core [14,15,16,17,18,19], many experimental material processing approaches exist: simple “one-pot” or complex “multi-step” ones; seed-mediated and non-seed-mediated routes; and surfactant-mediated and non-surfactant-mediated protocols [20]. However, within this processing richness, seed-mediated growth is certainly the most efficient one, since it allows better microstructural control and offers a stronger structural robustness. In practice, it proceeds by growing gold nanocrystals at the surface of a preformed core particle, using various reducing agents, such as hydroquinone [14,15,16], N,N-dimethylformamide (DMF) with PVP [17,18,19], L-ascorbic acid [21] and citric acid [22], all of them being weak reducing agents. They also allow gold salt redox reactions of high speed with an anisotropic gold crystal growth, which is useful for our purpose. Moreover, when combined (e.g., hydroquinone and citric/citrate), these weak reducing agents may present synergetic effects. For instance, hydroquinone produces Au^0^ nucleus preferentially oriented along with their crystallographic (111) direction, which may continue to grow within the same direction, favoring the production of branched tips, while citrate acts as a binding ligand contributing not only to the nucleation process but also to the improvement of the colloidal stability of the final products [22].

Besides, using this material engineering approach, it is crucial to maintain a high magnetization in the final architecture, to make Fe_3-x_O_4_-Au easily recoverable by a lab. magnet, when dispersed in an analyte solution. Hence, the use of highly magnetized magnetic multicores rather than single cores is required, as is keeping the diamagnetic gold shell thickness as low as possible. Moreover, iron oxide multicore particles often exhibit a superparamagnetic blocking temperature close to room temperature. In these conditions, they are able to align very quickly along an applied external magnetic field and to misalign also very quickly, when the magnetic field is off, becoming easily redispersible in a solution, to be used again, when coated by an anisotropic in shape gold shell, as SERS substrates.

So, in this paper, we report the preparation of such substrates and their use for the detection, in water, of thiram molecules at very low concentrations (thiram is a well-known pesticide [23]). The magneto-plasmonic building blocks were consecutively fabricated, starting from (i) the precipitation in polyol of multicore iron oxide nanoparticles (core of the final hybrids); then, (ii) their surface modification for the electrostatic attachment of ultrafine preformed negatively charged gold nanocrystals (seed particles); and finally, (iii) the use of the resulting hybrid particles as seeds to grow a rough gold shell thanks to the reduction of dissolved HAuCl_4_ salt in water with hydroquinone in the presence of citrates. Within these experimental conditions and by varying the weight ratio *r* between the hybrid seed particles and the gold salt precursor, a more or less continuous but anisotropic in shape gold layer can be formed around the iron oxide particles, leading to more or less efficient magneto-plasmonic nanostructures for SERS detection of analyte traces in water.

## 2. Experimental Section

### 2.1. Chemicals

Iron (III) chloride hexahydrate FeCl_3_.6H_2_O (98%), ethylene glycol anhydrous (EG, HO(CH_2_)_2_OH, 99.8%), poly(ethylene glycol) (PEG, M_W_ = 10,000 g mol^−1^), sodium acetate anhydrous (NaAc, CH_3_COONa, 99%), trisodium citrate (Na_3_C_6_H_5_O_7_, 99.9%), ammonium hydroxide (NH_4_OH, 25%) and gold (III) chloride trihydrate (HAuCl_4_.3H_2_O, 99.9%) were purchased from Sigma-Aldrich. (3-aminopropyl)triethoxysilane (APTES, C_9_H_23_NO_3_Si, 99%), sodium hydroxide (NaOH, 99%), hydroquinone (C_6_H_4_-1,4-(OH)_2_, 99%), tetrakis(hydroxymethyl) phosphonium chloride (THPC, [(CH_2_OH)_4_P]Cl, 80%) and absolute ethanol (98%) were purchased from Merck. Deionized water was used for all the preparations.

### 2.2. Characterization Tools

The crystalline structures of all the particles produced were checked by X-ray diffraction (XRD) using a Panalytical X’pert Pro diffractometer, working in the Bragg-Brentano reflection geometry and equipped with a multichannel X’celerator detector and a cobalt X-ray tube operating at 40 kV and 40 mA. The data were analyzed by the Rietveld method, using HighScore software (Panalytical) to determine the cell parameters and the average crystal sizes of the investigated crystalline phases. Detailed microstructural analysis was performed on aqueous suspensions sonicated for few minutes before deposition of a few drops on specific grids for transmission electron microscopy (TEM) or scanning electron microscopy (SEM) observations. TEM was carried out on a JEOL-100-CX II microscope operating at 100 kV, and SEM was carried out on a Zeiss Supra 40 microscope operating at 5 to 10 kV and equipped with an energy dispersive spectrometer (EDS). The recorded micrographs were analyzed using the ImageJ software, and a lognormal statistical law was applied to the inferred size distribution to determine the average particle size. Using the same suspensions, the hydrodynamic size distribution was determined by dynamic light scattering (DLS) using a Zeta Nanosizer Malvern instrument operating with a laser of 633 nm. This equipment also allows the determination of the zeta potentials (ζ) of the particles. The isoelectric point of these particles was determined through zeta potential measurements while varying the pH between 1 to 13, by adding HCl or NaOH to distilled water. The pH value was exactly measured by a JENCO Electronics Microcomputer pH-VISION 6071 pH-meter. UV-Visible absorption spectroscopy was also used to determine the plasmonic resonance band of all the produced gold-containing particles. A Perkin Elmer Lambda 1050 spectrometer equipped with a InGaAs and PbS 3-detector module was used for such a purpose, working in a transmission scheme. Finally, a Quantum Design SQUID-5T magnetometer was used to measure the thermal variation of the magnetization M(T) of the iron oxide-containing particles in the 2-330 K temperature range, in both field cooling (FC) and zero-field cooling (ZFC) conditions for an applied dc magnetic field of 200 Oe. Besides, the variation of the magnetization as a function of the magnetic field M(H) was also recorded by cycling the magnetic field between -50 and +50 kOe at room temperature.

### 2.3. SERS Substrates Preparation

**Figure 1:**
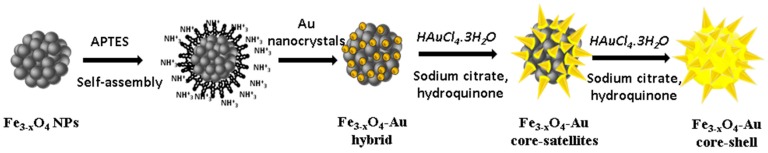
A schematic description of the different experimental steps involved in the production of star-shaped, Fe_3-x_O_4_-Au core-shell particles.

SERS substrates were prepared according a multi-step synthesis route involving separated preparation of iron oxide polycrystals and gold nanocrystals, attached together by electrostatic interactions. (Figure 1). As explained hereafter, the resulting nanostructures were used as seeds in a gold salt solution to grow a rough gold shell.

#### 2.3.1. Synthesis of Iron Oxide Particles and Their Amine-Functionalized Counterparts

**Figure 2:**
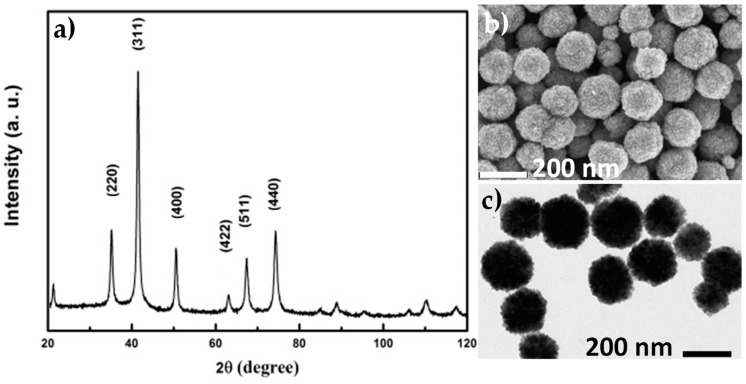
(**a**) XRD pattern of the magnetite-like core particles. (**b**,**c**) SEM and TEM representative micrographs of an assembly of these particles.

Submicrometer-sized magnetite-like (Fe_3-x_O_4_) particles (Figure 2) were synthesized by forced hydrolysis in polyol. In detail, 1.02 g of hexahydrate iron(III) chloride, 2.91 g of anhydrous sodium acetate and 1.03 g of polyethylene glycol (PEG) were dissolved in 40 mL of ethylene glycol (EG) under mechanical stirring in a triple-neck round-bottom flask. The mixture was then heated up to reflux (190 °C) for 12 h. Within these operating parameters, the precipitation of Fe_3-x_O_4_ proceeds in close-to-solvothermal conditions, offering to the resulting particles a high crystalline quality. After cooling down to room temperature, the particles were washed with ethanol and recovered by magnetic decantation before being dried in air at 60 °C. 

XRD pattern of the magnetite-like core particles matches very well with the ICDD card number 98-002-6410. The cell parameter and the average crystal size inferred from Rietveld refinements were found to be: a = 8.378(5) Å and L = 10(1) nm respectively. SEM and TEM representative micrographs of an assembly of these particles clearly evidence their polycrystalline nature and allow the measurement of their final average size, which is 180(20) nm.

**Figure 3:**
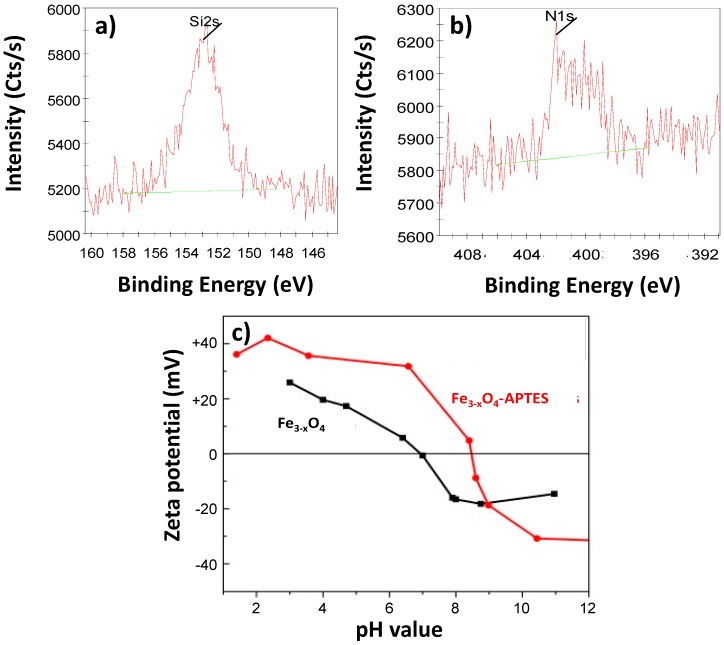
High resolution XPS spectra of (**a**) Si 2s (153 eV) and (**b**) N 1s signals (402 eV characteristic of protonated N) recorded on APTES grafted iron oxide particles, evidencing the signature of silane and ammonium species on the surface of the analyzed particles. (**c**) Zeta potential as a function of the pH measured on bare particles (isoelectric point (IEP) = 6.9) and the grafted to APTES ones (IEP = 8.9). The continuous line (red or black) is just a guide for the eye.

Dried particles were functionalized by APTES, a bifunctional ligand bearing, on one side, ethoxysilane groups able to be attached to iron oxide particles, and on the other side, an amino group able to be protonated at neutral pH conditions [24]. In a typical experiment, 225 mg of as-prepared Fe_3-x_O_4_ particles were dispersed in 500 mL of ethanol. Then, 2 mL of APTES were added into the reaction mixture under sonication for 30 min, and then under mechanical stirring. After that, 2 mL of ammonium hydroxide were added, and the mixture was continuously stirred for 6 h. The resulting powder was recovered by magnetic decantation after several ethanol washing steps. At the end, the surface modified particles were dried in an oven at 60 °C. The grafting was confirmed by X-ray photoelectron spectroscopy (XPS) and zeta potential measurement (Figure 3). Therefore, a net increase of the zeta potential, at neutral pH, from 0 to +30 mV, and a shift of the isoelectric point (IEP) from 6.9 to 8.9, were observed in agreement with the attachment of ammonium groups at the surface of the particles (Figure 3).

#### 2.3.2. Synthesis of Hybrid Seed Particles

**Figure 4:**
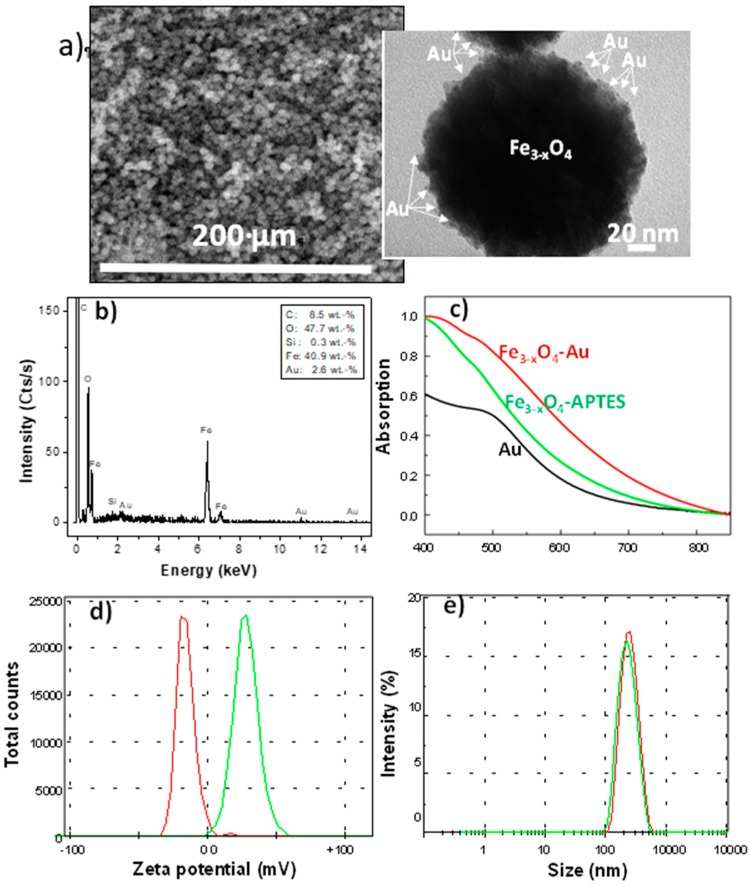
(**a**) SEM micrograph of an assembly of Fe_3-x_O_4_-Au hybrids and (**b**) its related EDS spectrum. In the insert, a TEM picture of one representative hybrid. (**c**) UV-Vis spectrum of gold colloid (black line) compared to those of Fe_3-x_O_4_ functionalized by APTES (green line) and then coated by gold satellites (red line). (**d**) Zeta potential distribution of the hybrids (red line) compared to that of Fe_3-x_O_4_ particles just coated with APTES (green line) when dispersed in deionized water. (**e**) Hydrodynamic size distribution as inferred from DLS measurement on the same suspensions (the size polydispersity index PDI was found to be 0.48 before grafting and 0.07 after grafting).

First, gold NPs of about 2 nm in size, which were negatively charged were prepared by the well-known Duff Baiker’s method [25]. In practice, 3 mL of an aqueous solution of sodium citrate (1 wt%) were dispersed in 80 mL of deionized water by sonication, and 0.4 mL of sodium hydroxide (1 M) was added to the mixture. About 1 mL of THPC (80 mM) was then dropped. After that, hydrochloride gold salt was continuously added to the reaction solution under stirring for 10 min. The as-produced gold single crystals, decorated at their surface with citrate ligands, were then electrostatically assembled to the positively charged polyol-made iron oxide polycrystals. The resulting hetero-nanostructures (Figure 4) were subsequently recovered by magnetic decantation and washed several times with water and then ethanol, before being dried in air at 60 °C. SEM micrograph of an assembly of Fe_3-x_O_4_-Au hybrids and its related EDS spectrum highlights the presence of 2.6 wt% of gold. In the insert, a TEM picture of one representative hybrid is given. Then, UV-Vis spectrum of gold colloid (black line) has been compared to those of Fe_3-x_O_4_ functionalized by APTES (green line) and then coated by gold satellites (red line); the plasmonic absorption at 500 nm confirms the attachment of gold nanocrystals in the hybrids. Zeta potential distribution (red line) of the hybrids compared to that of Fe_3-x_O_4_ particles just coated with APTES (green line) when dispersed in deionized water, points out the net decrease of the surface charge from +27 mV to −21 mV, evidencing gold nanocrystals’ attachment. Finally, hydrodynamic size distribution has been inferred from DLS measurement on the same suspensions, evidencing a slight size increase of hybrids (192 vs. 189 nm).

#### 2.3.3. Synthesis of the Star-Shaped Fe_3-x_O_4_‒Au Core-Shell Nanoparticles (NSs)

Star-shaped core-shell particles (NSs) were prepared by the reduction of HAuCl_4_ salt in the presence of the previously prepared hybrid seed particles. Typically, 0.9 mL of an aqueous solution of sodium citrate (1 wt%) was mixed to a given mass of auric salt (1 mg) and variable masses of seed particles (from 10 to 0.5 mg) in 8 mL of deionized water. The seed/HAuCl_4_ weight ratio, called *r*, was thus varied from a value of 10 to 8, 4, 2 and 0.5 in order to produce a series of composite particles of different chemical compositions and present different microstructures (from core-satellites to core-shell). The mixture was sonicated and stirred to ensure the dispersion of the seed particles in the reaction solution. About 3.3 mg of hydroquinone, the reducing agent, dissolved in 1 mL deionized water, was added to the previous mixture. The solution was continuously mechanically stirred for 10 min. The resulting particles were then separated by magnetic decantation and washed several times with deionized water.

### 2.4. Magnetically Assisted SERS Sensing

**Figure 5:**
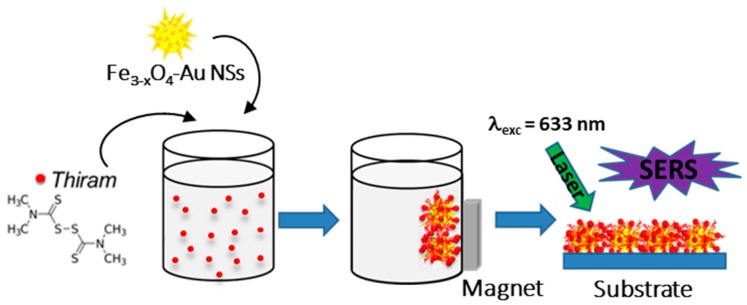
Concentration of the analytes on the star-shapped Fe_3-x_O_4_‒Au core-shell nanoparticles (NSs) before their collection by a magnet for SERS detection.

The as-prepared NSs were dispersed in 5 mL of analyte solution for 2 h to ensure the adsorption of the molecules on the surface of the particles. The analyte solution consisted of thiram diluted in deionized water at different concentrations, 10^−5^_,_ 10^−6^, 10^−7^ and 10^−8^ M. Subsequently, NSs were collected on the substrate surface by applying an external magnetic field. The substrates were taken out and rinsed with deionized water. The samples were dried in an oven for 10 min prior their analysis by SERS (Figure 5). 

Raman spectroscopy was conducted on a Jobin-Yvon LABRAM HR800 micro-spectrometer using a 633 nm laser source with a power of 64 µW. All spectra were taken with an exposure time of 3 s, and 10 accumulations and recorded within the 0–2280 cm^−1^ spectral range. The spectral resolution was less than 1.5 cm^−1^. For each sample, three SERS spectra at different positions of the substrate were performed and then averaged before being plotted.

## 3. Results and Discussion

### 3.1. Gold Shell Growth

**Figure 6:**
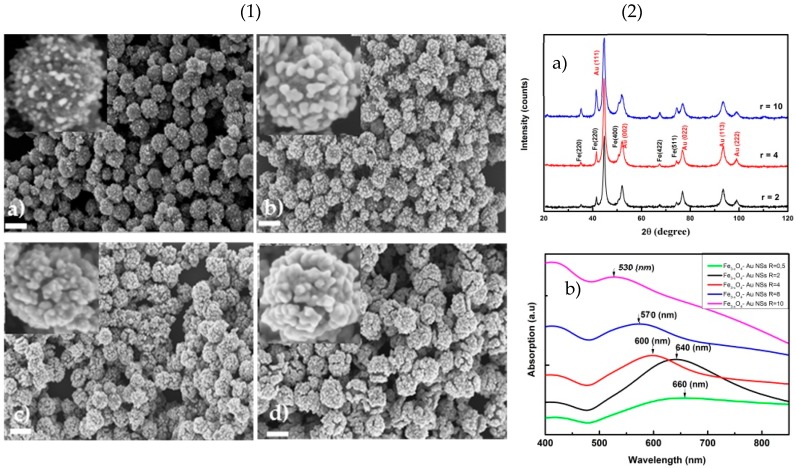
(**1**) SEM images of γ-Fe_2_O_3_‒Au NSs with different seed/HAuCl_4_ ratios of (**a**) *r* = 10, (**b**) *r* = 4, (**c**) *r* = 2, (**d**) *r* = 0.5. The scale bars are 200 nm in both figures; (**2**) XRD patterns (**a**) and UV-Vis spectra of Fe_3-x_O_4_‒Au NSs with different *r* values (**b**).

The growth of a continuous Au shell was accomplished by combining molecular self–assembly technique and colloidal growth chemistry. It took place around Au seeds present on the surface of Fe_3-x_O_4_ spherical polycrystals. While decreasing the *r* weight ratio between the seed particles and the gold salt reagent, the morphology of the resulting composite particles evolves from an incompletely coated core to a completely coated one. The growth mechanism for the formation of the gold shell can be depicted. At the beginning, the remaining Au^3+^ precursor is distributed in the reaction solution, while the number of gold seed particles is increased in direct proportion to *r* value. As a result, the gold crystals, that are formed on the surface of the magnetic seed particles, are larger. Figure 6(1) presents the SEM images of these nanocomposites for different *r* values (from 10 to 0.5). At the beginning, the morphology of the particles is mainly spherical, like that of the magnetic particles. Then, SEM images indicate that the gold nanocrystals are growing from the surface of the magnetic particles, forming larger and larger islands, which then coalesce. The inter-island distance decreases until they merge together, leading to a continuous gold coating, but also larger in size composite particles. The thickness of the gold layer is between 10 and 50 nm when the *r* value decreases from 10 to 0.5. It is interesting to notice that the gold shell is rough and that a star-shaped morphology is obtained for the smallest values of *r*. The reason is that both hydroquinone and sodium citrate interact synergistically to direct the shape-controlled growth of the seeds, preferentially from high-index faces (namely (111) surface), leading to the beginning of an anisotropic growth. It could be evidenced on the XRD patterns (Figure 6(2a) on which the high intensity diffraction peaks at 2ϴ positions of 44.7, 52.1, 76.8, 93.5 and 99.1° correspond to the (111), (002), (022), (113) and (222) planes of face-centered cubic gold phase. The highest diffraction peak corresponded to the highest energy facet (111) and permitted the preferential deposition along the growth direction of Au^0^. In addition, several peaks of low intensity appear to correspond to the cubic spinel iron oxide pattern without any shift, in agreement with the fact that gold growth does not affect the chemical nature or the crystallographic structure of the starting magnetic polycrystals. Of course, as the gold shell becomes thicker and rougher as the intensity of the gold diffraction peaks in the composite patterns becomes higher, the intensity of the iron oxide diffraction peaks becomes lower. 

To complete these microstructural investigations, the UV-Visible spectra of the aqueous suspensions of all the produced composite particles were recorded. Interestingly, all the collected spectra exhibit a strong absorption related to the plasmonic properties of their gold component (Figure 6(2b). The maximum absorption wavelength is directly dependent on the morphological properties of the samples. Additionally, by comparing the spectra to each other, while decreasing their *r* value, it appears that their plasmonic band is red-shifted from 530 to 680 nm when *r* decreases from 10 to 0.5. The origin of this shift is clearly the evolution of the morphology of the composite particles—the gold branched ones, absorbing much more in the red region than the more isotropic ones (gold is fully covering the core component). Hence, varying the thickness of the gold shell leads to different wavelength absorption peaks. At ratio values of 10 and 4, the particles exhibit the plasmonic feature of individual Au NPs on the iron oxide surface. The junctions and ends of tips produce a rough surface for SERS activity. 

At the end, the colloidal stability of all the produced composite particles was checked. In practice, DLS measurements were performed in water, and the hydrodynamic size distribution of each prepared colloid was measured. A monomodal hydrodynamic size distribution with relatively low PDI values was observed in all the cases. The average hydrodynamic diameters were found to be larger than those inferred from SEM observations, and they were also found to be dependent on the nominal *r* ratio value (see Figure S1 and Table S1 in the Figure S1 and supporting information section: the diameter increases when *r* decreases. Typically, it ranged between 225.4 and 422.2 nm for the engineered magneto-plasmonic composite particles, the highest value corresponding to the smallest *r* value (*r* = 0.5), and in reverse, the lowest value corresponding to the highest *r* value (*r* = 10). 

**Figure 7:**
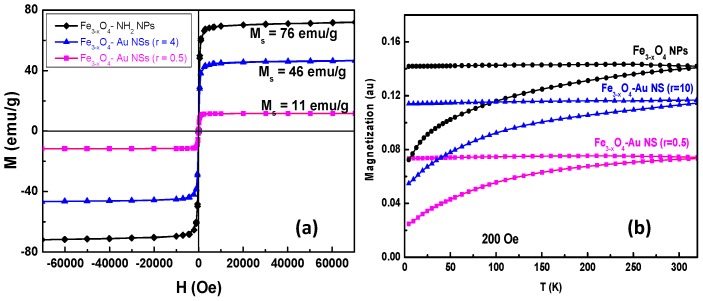
(**a**) Magnetization curves measured at 300 K; (**b**) ZFC- and FC-M(T) curves measured at 200 Oe on γ-Fe_2_O_3_-NH_2_ NPs and γ-Fe_2_O_3_‒Au NSs (*r* = 0.5 and 4). The continuous line is just a guide for the eye.

Then, the magnetic properties of all the produced iron oxide particles and their related star-shaped gold-based composites were investigated using a SQUID magnetometer. The measurements were conducted on the particles in their powder forms. The variation of their magnetization as a function of the magnetic field M(H) was thus measured at 5 and 300 K, as well the field-cooled (FC) and zero-field-cooled (ZFC) thermal variations of their magnetization. The recorded plots are presented in Figure 7 only for the particles corresponding to the largest iron oxide core size, since they exhibit higher magnetization at room temperature and higher blocking temperature. 

The M(H) curves recorded at room temperature (RT), on both bare iron oxide particles and their star-shaped gold-based composite particles, are all characteristic of soft ferrimagnets. They exhibit a hysteresis feature with a non-zero but very low remanence (10 emu g^−1^) and coercivity (62 Oe). These curves appear as almost completely reversible, without significant remanence and coercivity (Figure 7a). These results suggest that there is no significant magnetic interaction between the particles, remaining after removing the external magnetic field: this should facilitate their further dispersion in liquid media. The saturation magnetization of pristine iron oxide is found to be 76 emu g^−1^, while it is 46.5 emu g^−1^ for the composite particles, corresponding to NSs prepared starting from a *r* value of 4. This magnetization decrease is of course due to the diamagnetic contribution of the gold shell. The M(T) curves recorded on the two same samples are characteristic of a superparamagnetic behavior but with a blocking temperature significantly higher than RT (Figure 7b). T_B_ is usually dependent on the magnetic particle size, which is here large enough to block their magnetization over thermal fluctuation at RT. It is also dependent on the mutual dipolar interactions which are here particularly attenuated in the composite sample due to the presence of the gold shell.

### 3.2. Toward Magnetically Assisted SERS Sensing

SERS signals are located on the surfaces of metal nanostructures with strong electromagnetic fields mainly localized in spatially narrow regions such as tips, edges and vertices. The γ-Fe_2_O_3_-Au composite particles, with a magnetic core of 180 nm in diameter and a gold shell thickness of 20 nm (corresponding to a *r* experimental synthesis parameter of 4), are the most appropriate to be used as SERS substrates: they exhibit a SPR band around λ = 600 nm close to the wavelength of the laser source and a RT magnetization of 46 emu g^−1^, high enough to make their magnetic collection easy-to-achieve in a water suspension.

**Figure 8:**
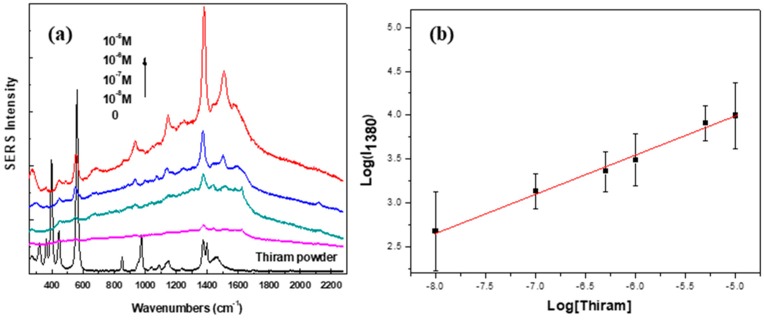
(**a**) SERS spectra of thiram, in solutions of various concentrations on Fe_3-x_O_4_-Au NSs; (**b**) plot of log of SERS intensity at 1380 cm^−1^ as a function of log of thiram concentrations in diluted water. Each point in the plot represents the average value of three random measurements on γ-Fe_2_O_3_-Au NSs. Errors bars correspond to standard deviations from these 3 experiments. The red line is the linear fitting curve based on the average values. Slope (0.43 ± 0.01); intercept (6.12 ± 0.12); *R^2^* = 0.99.

In practice, analyte aqueous solutions containing a given contaminant (namely, thiram) were prepared by dissolving a certain amount of thiram (balance accuracy = 10^−5^ g) in 2000 mL of water (leading to an error of about 2 × 10^−8^ mol L^−1^ on the concentration of thiram); then, the selected magnetic-plasmonic particles were dispersed, at different concentrations, to form stable colloids. They were then collected onto SERS substrate by applying an external magnetic field. Homogeneously distributed aggregates were formed spontaneously on the substrate wafer after water evaporation. The SERS measurements were carried out to detect thiram on the substrate for different solute concentrations: 10^−5^ M, 10^−6^ M, 10^−7^ M and 10^−8^ M. According to theoretical calculations, molecules located at the junction between aggregated plasmonic particles can enhance SERS signal over 10 orders of magnitude. Raman spectra of thiram randomly collected from different positions on the substrate were recorded and plotted in Figure 8 and compared to pure solid thiram. 

Interestingly, the characteristic peaks of thiram, positioned at 560, 934, 1147 and 1380 cm^−1^, corresponding to the ν(S-S) stretching mode; ν(CH_3_N) and ν(C=S) stretching mode; ρ(CH_3_) rocking mode and ν_as_(CN) stretching mode; and δ_s_(CH_3_) deformation mode and ν(CN) stretching mode, respectively, are clearly evidenced in the spectra collected on the engineered substrates (Figure 8a). These results indicate that thiram can be well detected using the present magneto-plasmonic particles, even at solute concentrations as low as 10^−8^ M. This result clearly evidences that γ-Fe_2_O_3_-Au based substrates are highly sensitive and promising for the detection of target molecules. 

Moreover, focusing on the most intense thiram band, centered at 1380 cm^−1^, the plot of log of its intensity as a function of log of thiram concentration (Figure 8b) shows a quantification region (between 10^−8^ and 10^−5^ M) with a linear relationship, which can be expressed by:

log(I_1380_) = 0.43 × log[thiram] + 6.12 (1)

For a thiram concentration higher than 10^−5^ M, the Raman intensity is constant or slightly decreased, which may be due to the saturation of the adsorption of thiram on the SERS hot spots. The analytical enhancement factors (AEF) are estimated from:

AEF = (I_SERS_/C_SERS_)/(I_RS_/C_RS_) (2)

where I_SERS_ is the SERS signal at C_SERS_ concentration of thiram, and I_RS_ is the Raman signal under non-SERS conditions at C_RS_ concentration of thiram. By applying this equation and the previous one, an AEF of 2 × 10^5^ was found for the present engineered magnetic SERS substrate. This is a pretty good value regarding the existing literature. Among the most relevant studies closest to ours, we can mention that Han et al. reported the synthesis of Fe_3_O_4_-Au core-satellite and core-shell nanocomposites prepared by thermal decomposition followed by seed-mediated growth; these latter have been used to detect thiram on apple peel and demonstrated a better sensitivity for SERS detection of this pesticide than core-satellite equivalents. AEF of core-shell nanoparticles have been evaluated, using the same method, to be 3.76 × 10^5^, nearly fifteen times higher than that of core-satellite systems (2.56 × 10^5^) [26]. Other studies dealing with hetero-nanostructures composed of iron oxide and silver components, and prepared by different synthetic routes, present experimental AEF values of ca. 2 × 10^8^ while theoretical calculated ones were found to be 5–6 × 10^8^ [27,28]. By the way, all the authors underlined the difficulties to estimate this AEF but concluded that their systems were suitable for portable Raman spectrometers for rapid detection. Besides, a main drawback of several systems reported in the literature could be the production of individual gold particles during the coating process that could enhance the AEF. In our case, the enhancement factor could be lower since we recovered the magnetic hybrids through magnetic decantation; hence the gold particles were removed in the supernatant. Indeed, a major advantage of the polyol process is the very good control over the crystallinity leading to high magnetizations, close to the bulk. However, from a structural point of view, the objective would be to sharpen the absorption peak more (<150 nm) in the (N)IR region to improve the sensing performance [29]. 

Therefore, our value clearly evidences that the magneto-plasmonic hybrid particles presented in this paper, synthesized by the polyol process combined to seed-mediated growth, may be considered as good candidates for the SERS detection of pesticides. 

## 4. Conclusions

We successfully developed a new, efficient chemical strategy to prepare star-shaped Fe_3-x_O_4_-Au NSs for magnetically assisted SERS sensing. In practice, polyol-made magnetite-like 180 nm sized polycrystals, functionalized by APTES, were electrostatically interacted with citrate-made 2 nm sized gold single crystals. The resulting hybrids were used as seeds to grow—in a fresh auric solution, containing hydroquinone—gold islands, which progressively became adjacent and branched. In the end, for an optimized nominal seeds/HAuCl_4_ weight ratio, the desired NSs presented a magnetization of 46.5 emu g^−1^ at room temperature, and plasmonic resonance absorption at around 650 nm, making them valuable for magnetic separation and SERS application. Dispersed in a fresh very diluted (10^−8^ M) analyte solution of thiram, they were then collected by a magnet deposited on a silicon sheet and successfully used as SERS substrates for thiram trace detection.

## Supplementary Materials

The following are available online at https://www.mdpi.com/2079-4991/10/2/294/s1, Figure S1: DLS measurements of FexO4-Au NSs using hydroquinone as reducing agent with different r ratios: a) FexO4-NH2, b) r = 10, c) r = 4, d) r = 2, and e) r = 0.5; Table S1: Summary of the main data: size (SEM), hydrodynamic diameter, PDI and SPR of FexO4-Au NSs using hydroquinone as reducing agent with different r ratios of 10, 4, 2 and 0.5.

## References

[1] Cai Q., Hu F., Lee S.-T., Liao F., Li Y., Shao M. (2015). Controllable Fe_3_O_4_/Au substrate for surface-enhanced infrared absorption spectroscopy. Appl. Phys. Lett..

[2] Gao Q., Zhao A., Guo H., Chen X., Gan Z., Tao W., Zhang M., Wu R., Li Z. (2014). Controlled synthesis of Au-Fe_3_O_4_ hybrid hollow spheres with Excellent SERS activity and catalytic properties. Dalton Trans..

[3] Guo Q.H., Zhang C.J., Wei C., Xu M.M., Yuan Y.X., Gu R.A., Yao J.L. (2016). Controlling dynamic SERS hot spots on a monolayer film of Fe_3_O_4_@Au nanoparticles by a magnetic field. Spectrochim. Acta A.

[4] Ding G., Xie S., Zhu Y., Liu Y., Wang L., Xu F. (2015). Graphene oxide wrapped Fe_3_O_4_@Au nanohybrid as SERS substrate for aromatic dye detection. Sens. Actuators B.

[5] Xie Y., Chen T., Guo Y., Cheng Y., Qian H., Yao W. (2019). Rapid SERS detection of acid orange II and brilliant blue in food by using Fe_3_O_4_@Au core–shell substrate. Food Chem..

[6] Chen Y., Zhang Y., Kou Q., Liu Y., Han D., Wang D., Chen L. (2018). Enhanced Catalytic Reduction of 4-Nitrophenol Driven by Fe_3_O_4_-Au Magnetic Nanocomposite Interface Engineering: From Facile Preparation to Recyclable Application. Nanomaterials.

[7] Sun Z., Du J., Duan F., He K., Jing C. (2019). Simulation and synthesis of Fe_3_O_4_-Au satellite nanostructures for optimised surface-enhanced Raman scattering. J. Mater. Chem. C.

[8] Schatz G.C., Young M.A., Van Duyne R.P. (2006). Electromagnetic mechanism of SERS. Surface-Enhanced Raman Scattering.

[9] Qiu Y., Deng D., Deng Q., Wu P., Zhang H., Cai C. (2015). Synthesis of magnetic Fe_3_O_4_/Au hybrids for sensitive sers detection of cancer cells at low abundance. J. Mater. Chem. B.

[10] Lou L., Yu K., Zhang Z., Huang R., Zhu J., Wang Y., Zhu Z. (2012). Dual-mode protein detection based on Fe_3_O_4_/Au hybrid nanoparticles. Nano Res..

[11] Li F., Yu Z., Zhao L., Xue T. (2016). Synthesis and application of homogeneous Fe_3_O_4_C core/Au shell nanoparticles with strong SERS effect. RSC Adv..

[12] Zhou X., Xu W., Wang Y., Kuang Q., Shi Y., Zhong L., Zhang Q. (2010). Fabrication of cluster/shell Fe_3_O_4_/Au nanoparticles and application in protein detection via a SERS method. J. Phys. Chem. C.

[13] Pang Y., Wan N., Shi L., Wang C., Sun Z., Xiao R., Wang S. (2019). Dual-recognition surface-enhanced Raman scattering (SERS) biosensor for pathogenic bacteria detection by using vancomycin-SERS tags and aptamer-Fe_3_O_4_@Au. Anal. Chim. Acta.

[14] Zhou H., Choi S.I., Zou F., Oh S., Kim J.E., Hwang D.Y., Lee J. (2014). Cytotoxicity and gene expression in sarcoma 180 cells in response to spiky magnetoplasmonic supraparticles. ACS Appl. Mater. Interfaces.

[15] Zhou H., Kim J.-P., Bahng J.H., Kotov N.A., Lee J. (2014). Self-assembly mechanism of spiky magnetoplasmonic supraparticles. Adv. Funct. Mater..

[16] Li C., Chen T., Ocsoy I., Zhu G., Yasun E., You M., Wu C., Zheng J., Song E., Huang C.Z. (2014). Gold-coated Fe_3_O_4_ nanoroses with five unique functions for cancer cell targeting, imaging and therapy. Adv. Funct. Mater..

[17] Espinosa A., Bugnet M., Radtke G., Neveu S., Botton G.A., Wilhelm C., Abou-Hassan A. (2015). Can magneto-plasmonic nanohybrids efficiently combine photothermia with magnetic hyperthermia?. Nanoscale.

[18] Quaresma P., Osório I., Dória G., Carvalho P.A., Pereira A., Langer J., Araújo J.P., Pastoriza-Santos I., Liz-Marzán L.M., Franco R. (2014). Star-shaped magnetite@gold nanoparticles for protein magnetic separation and SERS detection. RSC Adv..

[19] Reguera J., Jimenez de Aberasturi D., Winckelmans N., Langer J., Bals S., Liz-Marzan L.M. (2016). Synthesis of janus plasmonic-magnetic, star-sphere nanoparticles, and their application in SERS detection. Faraday Discuss..

[20] Nguyen T.T., Mammeri F., Ammar S. (2018). Iron oxide and gold based magneto-plasmonic nanostructures for medical applications: A review. Nanomaterials.

[21] Kwizera E.A., Chaffin E., Shen X., Chen J., Zou Q., Wu Z., Gai Z., Bhana S., O’Connor R., Wang L. (2016). Size- and shape-controlled synthesis and properties of magnetic-plasmonic core-shell nanoparticles. J. Phys. Chem. C.

[22] Li J., Wu J., Zhang X., Liu Y., Zhou D., Sun H., Zhang H., Yang B. (2011). Controllable synthesis of stable urchin-like gold nanoparticles using hydroquinone to tune the reactivity of gold chloride. J. Phys. Chem. C.

[23] Kang J.S., Hwang S.Y., Lee C.J., Lee M.S. (2002). SERS of dithiocarbamate pesticides adsorbed on silver surface; Thiram. Bull. Korean Chem. Soc..

[24] Galindo-Gonzalez C., Gantz S., Ourry L., Mammeri F., Ammar-Merah S., Ponton A. (2014). Elaboration and Rheological Investigation of Magnetic Sensitive Nanocomposite Biopolymer Networks. Macromolecules.

[25] Duff D.G., Baiker A., Edwards P.P. (1993). A new hydrosol of gold clusters. 1. Formation and particle size variation. Langmuir.

[26] Han D., Li B., Chen Y., Wu T., Kou Y., Xue Y., Chen L., Liu Y., Duan Q. (2019). Facile synthesis of Fe_3_O_4_@Au core–shell nanocomposite as a recyclable magnetic surface enhanced Raman scattering substrate for thiram detection. Nanotechnology.

[27] Wang C., Li P., Wang J., Rong Z., Pang Y., Xu J., Dong P., Wang S. (2015). Polyethylenimine-interlayered core–shell–satellite 3D magnetic microspheres as versatile SERS substrates. Nanoscale.

[28] Wang C., Wang J., Li P., Jia X., Ma Q., Xiao R., Wang S. (2016). Sonochemical synthesis of highly branched flower-like Fe_3_O_4_@SiO_2_@Ag microcomposites and their application as versatile SERS substrates. Nanoscale.

[29] Ovejero J.G., Yoon S.J., Mayoral A., Gao X., O’Donnell M., Garcia M.A., Herrasti P., Hernando A. (2018). Synthesis of hybrid magneto-plasmonic nanoparticles with potential use in photoacoustic detection of circulating tumor cells. Michrochim. Acta.

