# Supplementary material for: Star-Shaped Fe3-xO4-Au Core-Shell Nanoparticles: From Synthesis to SERS Application
Source: Nanomaterials (Basel). 2020 Feb 10;10(2):294. doi: 10.3390/nano10020294 (PMC7075140; doi:10.3390/nano10020294)
Supplement: Supplementary file 1 [file nanomaterials-10-00294-s001.pdf]

nanomaterials

Supplementary

Star-Shaped $Fe_{3-x}O_4$-Au Core-Shell Nanoparticles: From Synthesis to SERS Application

Thi Thuy Nguyen [1,2,3,4,*], Stephanie Lau-Truong [1], Fayna Mammeri [1,*] and Souad Ammar [1]

1 Université de Paris, ITODYS, CNRS, UMR 7086, 15 rue J-A de Baïf, 75013 Paris, France; stephanie.lau@univ-paris-diderot.fr (S.L.-T.); ammarmer@univ-paris-diderot.fr (S.A.)
2 Department of Advanced Materials Science and Nanotechnology, University of Science and Technology of Hanoi, Vietnam Academy of Science and Technology, 18 Hoang Quoc Viet, 10000 Hanoi, Vietnam
3 Graduate University of Science and Technology, Vietnam Academy of Science and Technology, 18 Hoang Quoc Viet, Cau Giay, 10000 Hanoi, Vietnam
4 Institute of Physics, Vietnam Academy of Science and Technology, 18 Hoang Quoc Viet, Cau Giay, 10000 Hanoi, Vietnam
* Correspondence: thuynt@iop.vast.ac.vn (T.T.N.); fayna.mammeri@u-paris.fr (F.M.)

Received: 27 December 2019; Accepted: 06 February 2020; Published: date

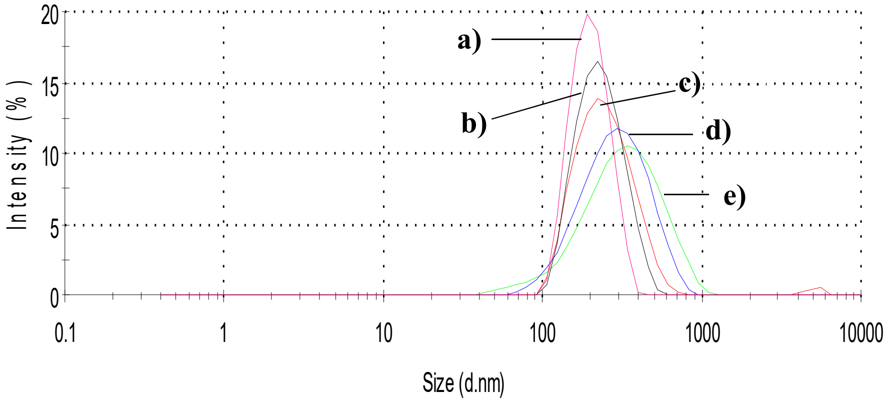

Figure S1. DLS measurements of Fe_xO_4-Au NSs using hydroquinone as reducing agent with different *r* ratios: a) Fe_xO_4-NH_2, b) *r* = 10, c) r = 4, d) *r* = 2, and e) r = 0.5.

Table S1. Summary of the main data: size (SEM), hydrodynamic diameter, PDI and SPR of Fe_xO_4-Au NSs using hydroquinone as reducing agent with different *r* ratios of 10, 4, 2 and 0.5.

Sample	d_{SEM} (nm)	d_{DLS} (nm)	PDI	λ (nm)
Fe40-NH_2	180	189.0	0.07	-
Fe40-AuR10	210	225.4	0.12	530
Fe40-AuR4	240	265.7	0.24	600
Fe40-AuR2	260	324.5	0.30	650
Fe40-AuR0.5	280	422.2	0.45	680
